# A Systematic Review on Whether an Association Exists Between Adolescent Obesity and Idiopathic Intracranial Hypertension

**DOI:** 10.7759/cureus.28071

**Published:** 2022-08-16

**Authors:** Sana Zafar, Venkatesh Panthangi, Adrienne R Cyril Kurupp, Anjumol Raju, Gaurav Luthra, Mahrukh Shahbaz, Halah Almatooq, Paul Foucambert, Faith D Esbrand, Safeera Khan

**Affiliations:** 1 Internal Medicine, California Institute of Behavioral Neurosciences & Psychology, Fairfield, USA; 2 Pediatrics, California Institute of Behavioral Neurosciences & Psychology, Fairfield, USA; 3 Dermatology, California Institute of Behavioral Neurosciences & Psychology, Fairfield, USA

**Keywords:** headache, overweight, ptcs, vision loss, lumbar puncture, papilledema, adolescent obesity, obesity, pseudotumor cerebri, idiopathic intracranial hypertension

## Abstract

Pseudotumor cerebri syndrome (PTCS)/idiopathic intracranial hypertension (IIH) is a clinical presentation appertaining to signs/symptoms of raised intracranial pressure, like headache and papilledema. It is an uncommon but clinically significant cause of morbidity such as permanent vision loss. It is crucial to understand if idiopathic intracranial hypertension (IIH) is on the rise in adolescents, it is probably due to the rising prevalence of obesity worldwide. Our study aimed to find an association between obesity and IIH in adolescents. We utilized Preferred Reporting Items for Systematic Review and Meta-Analysis 2020 (PRISMA) guidelines to run this systematic review. Many publications related to the topic in the discussion were scrutinized through a comprehensive database search. We filtered them down to a final count of 10 articles after utilizing our inclusion/exclusion criteria and assessing the quality of work. In these final papers, we identified several possibilities to explain the link between obesity and IIH in adolescents. Overweight and obese adolescents were found to have a significantly increased risk of IIH development, with a more severe clinical picture seen in morbidly obese female patients.

## Introduction and background

Idiopathic intracranial hypertension (IIH), also called primary pseudotumor cerebri syndrome (PTCS), is an umbrella term for the collection of symptoms caused by increased intracranial pressure (ICP) with normal cerebrospinal fluid (CSF) constituents and brain parenchyma [1]. It affects young, overweight adolescents with signs and symptoms of headache, nausea/vomiting, or visual symptoms such as transient vision loss, visual field impairment, photopsia, double vision, and eye pain [2]. More symptoms of IIH may include pulse-synchronous tinnitus and shoulder/arm pain [2,3]. Impaired visual acuity, loss of visual field, and papilledema are also among the ophthalmic signs of IIH. Sixth or seventh nerve palsies may also be present in up to 30% of patients resulting in permanent loss of vision [2]. The signs/symptoms of IIH are variable among the pediatric age group. Adolescents can describe visual symptoms, such as transient loss of vision, "shimmering lights with colored centers," or sometimes photophobias [4]. Headache is the most common symptom among adolescents with IIH, which has been reported in 62%-91% of patients [5-7]. There have been instances where IIH was reported without symptoms of headache in children, possibly because of the very young age of a child to report or the chance that headache was truthfully not present [8-11]. IIH cases in adolescents without the symptom of headache are seen to have more neural signs, and presentation with loss of vision is related to a worse prognosis [6]. Furthermore, in adolescent cases of IIH, the presentation can be seen as a posterior fossa lesion with facial asymmetry, stiff neck, and wry neck [12].

PTCS is an avoidable cause of loss of vision, and it is essential to diagnose it early and initiate appropriate treatment. This disorder can be seen in all age groups and genders, although it occurs more frequently in obese childbearing age females. PTCS is diagnosed at increasing rates, probably because of the ever-increasing obesity epidemic worldwide and awareness about the disease [13].

**Figure 1:**
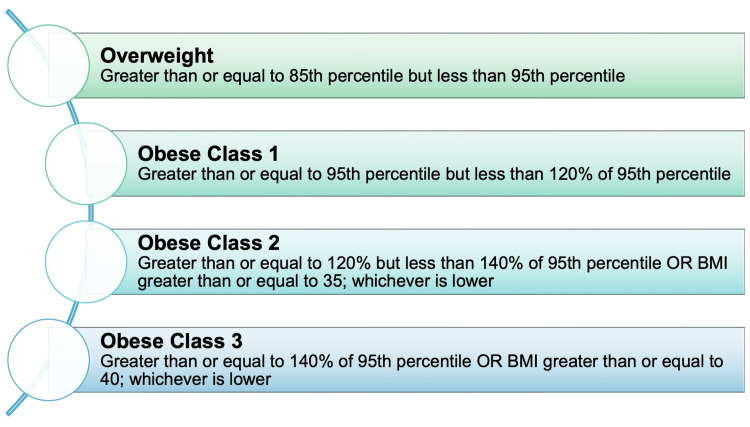
Weight properties BMI: body mass index. Figure created by Sana Zafar.

Cases of obesity among adolescents in the United States have increased dramatically along with the prevalence of weight-related diseases. Body mass index (BMI) is characterized as weight in kilograms divided by height in meters squared. Measuring BMI is an easy, effective way to identify adolescents at risk of weight-related diseases [14,15]. The characteristics of overweight, obesity, severe obesity, and extreme obesity are discussed in Figure 1 [16].

The few studies that have assessed the relationship between pediatric IIH and obesity have yielded conflicting results [10,16-18]. Few studies proposed that obesity is just a risk factor for IIH in children at puberty [17]. All had notable methodological restrictions, including illustrative case series design with information on obesity on cases only, limited sample sizes (15-50), and referral center bias [5,10,16-18]. Risk estimates were not reported by any of these studies and were not imperative when counseling overweight/obese patients to prevent IIH. This review aimed to recognize the risk factors for pediatric IIH and estimate the expanse of the association between overweight, moderate, and extreme childhood obesity and the risk of pediatric IIH.

## Review

Method

We reported the findings in accordance with the Preferred Reporting Items for Systematic Review and Meta-Analysis (PRISMA) principles and criteria. The systematic review was conducted according to these standards and principles [19].

Search Sources/Search Strategy

PubMed, Medline, PubMed Central (PMC), and Science Direct were utilized as main research literature search engines and databases. The search was managed using keywords and Medical Subject Headings (MeSH) criteria on April 15, 2022, which resulted in a vast number of papers signifying a link between obesity and idiopathic intracranial hypertension in adolescents. "Obesity," "idiopathic intracranial hypertension," and "adolescents" were the main terms used in the literature search. Furthermore, the MeSH strategy utilized in PubMed and PMC for the keywords mentioned above was the AND function of the three statements mentioned in Table 1.

**Table 1:** MeSH query strings MeSH: Medical Subject Headings. Table created by Sana Zafar.

Statement 1	Statement 2	Statement 3
(“Obesity/cerebrospinal fluid”[Mesh] OR “Obesity/complications”[Mesh] OR “Obesity/diagnosis”[Mesh] OR “Obesity/etiology”[Mesh] OR “Obesity/metabolism”[Mesh] OR “Obesity/physiopathology”[Mesh])	(“Pseudotumor Cerebri/anatomy and histology”[Mesh] OR “Pseudotumor Cerebri/cerebrospinal fluid”[Mesh] OR “Pseudotumor Cerebri/classification”[Mesh] OR “Pseudotumor Cerebri/diagnosis”[Mesh] OR “Pseudotumor Cerebri/etiology”[Mesh] OR “Pseudotumor Cerebri/physiopathology”[Mesh])	(“Adolescent/complications”[Mesh] OR “Adolescent/diagnosis”[Mesh] OR “Adolescent/etiology”[Mesh] OR “Adolescent/growth and development”[Mesh] OR “Adolescent/metabolism”[Mesh])

The total number of papers revealed in PubMed/Medline decreased to 79 from 188 after the initial search. Furthermore, the Science Direct database search resulted in 820 papers, narrowed down to 345. Other databases and search engines, such as MDPI, Cochrane library, and Web of Science, were not able to find any papers relevant to our inquiry. Gray literature was removed from this analysis in order to yield a good systematic review.

Eligibility Criteria

The literature papers included in this systematic review were chosen according to the following criteria:

Inclusion criteria: Inclusion criteria were as follows: articles published within the last 10 years, including full-text papers only, patients with characteristics that are relevant to obesity, clinical and imaging findings consistent with IIH in the adolescent age group, papers in English language or have an English translation available, and peer-reviewed and mixed study types.

Exclusion criteria: Exclusion criteria were as follows: papers outside the adolescent age group, gray literature, and unpublished papers.

Article Screening and Assess for Eligibility

For PubMed/PMC, we extracted articles to EndNote Citation Manager, where duplicates were removed. For other databases, we used automation filter tools. Records were then searched by title or abstract to sort out those considered unqualified. We then used our inclusion and exclusion criteria to comb through the remaining data. The remaining papers were assessed for eligibility using quality assessment tools.

Results

Search Outcome

**Figure 2:**
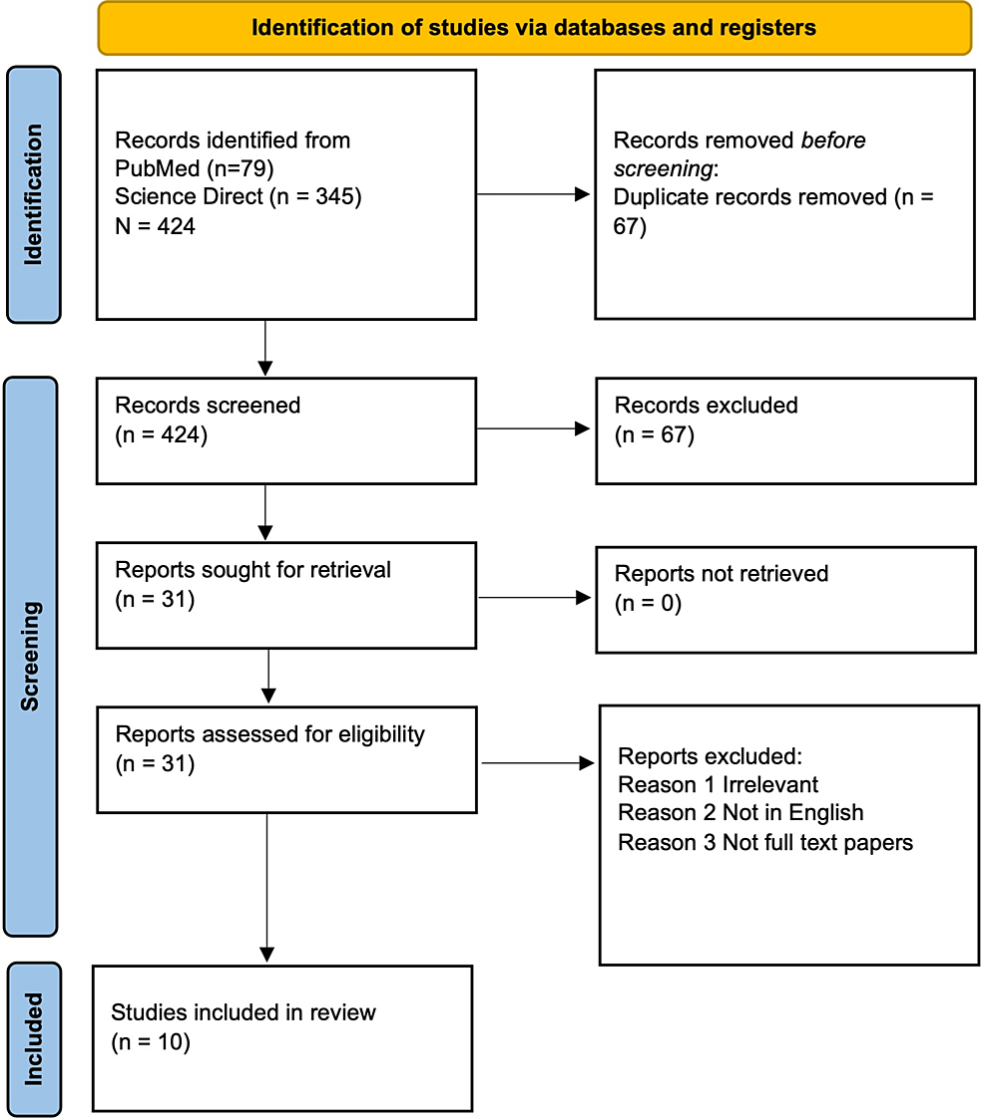
PRISMA 2020 flow diagram for systematic review PRISMA: Preferred Reporting Items for Systematic Review and Meta-analysis, PMC: PubMed Central.

After the initial screening, we narrowed down around 424 items out of the identified papers. Sixty-seven duplicate papers were removed approximately. After that, publications were refined by title/abstract, and we eliminated few studies owing to a lack of full-text papers and/or unrelated articles. Finally, after assessing 31 items for eligibility, we included a total of 10 papers in our review. Figure 2 is the flow chart for article selection formulated on PRISMA.

Quality Assessment

This systematic review comprised two study types: case reports and observational studies (cross-sectional, cohort, and case-control studies). The Joanna Briggs Institute (JBI) check tool for case reports was used to evaluate the quality of the case reports. We evaluated the observational studies (cross-sectional, cohort, and case-control studies) for a quality check using the Joanna Briggs Institute (JBI) checklist for cross-sectional, cohort, and case-control studies, respectively. This literature review involved all articles with a 60% or above score. The findings are summarized in Tables 2-5.

**Table 2:** Joanna Briggs Institute (JBI) critical appraisal checklist for case report Adapted from Ybarra et al. 2020 [20].

Study	Ybarra et al. 2020 [20]
1. Were the patient's demographic characteristics clearly described?	Yes
2. Was the patient's history clearly explained and presented as a timeline?	Yes
3. Was the current presenting clinical condition of the patient clearly explained?	Yes
4. Were diagnostic tests/assessment methods and the results clearly described?	Yes
5. Was the intervention(s)/treatment procedure(s) clearly described?	Yes
6. Was the post-intervention clinical condition elaborated?	Yes
7. Were adverse events (harms)/unanticipated events identified and described?	No
8. Does the case report advise takeaway lessons?	No
9. Quality evaluation?	Include

**Table 3:** Joanna Briggs Institute (JBI) critical appraisal checklist for a cross-sectional study Adapted from Brara et al. 2012 [21].

Study	Brara et al. 2012 [21]
1. Were the inclusion criteria in the sample clearly defined?	Yes
2. Were the study subjects and the setting described in detail?	Yes
3. Was the exposure measured in a reliable and valid way?	Yes
4. Were standard criteria used for the measurement of the condition?	Yes
5. Were confounding factors identified?	Yes
6. Were strategies for dealing with confounding factors stated?	Yes
7. Were the outcomes measured in a reliable and valid way?	Yes
8. Was appropriate statistical analysis used?	Yes
9. Overall evaluation?	Include

**Table 4:** Joanna Briggs Institute (JBI) critical appraisal checklist for case-control studies Adapted from Stiebel-Kalish et al. 2014 [22].

Study	Stiebel-Kalish et al. 2014 [22]
1. Were both the groups comparable other than the presence of disease in cases vs the absence of disease in controls?	Yes
2. Were cases and controls matched appropriately?	Yes
3. Were the same criteria used to identify cases and controls?	Yes
4. Was exposure measured in a standard, reliable and valid way?	Yes
5. Was exposure measured the same way for cases and controls?	Yes
6. Were confounding factors identified?	Unclear
7. Were strategies for dealing with confounding factors stated?	Unclear
8. Were outcomes assessed in a standard, reliable and valid way for cases and controls?	Yes
9. Was the exposure period long enough to be meaningful?	Yes
10. Was appropriate statistical analysis used?	Yes
11. Quality appraisal	Include

**Table 5:** Joanna Briggs Institute (JBI) critical appraisal checklist for cohort studies Adapted from Per et al., 2013 [23]; Tibussek et al., 2013 [24]; Değerliyurt et al., 2014 [25]; Bursztyn et al., 2014 [26]; Sheldon et al., 2016 [27]; Matthews et al., 2017 [28]; and Mahajnah et al., 2020 [29]. N/A: not applicable.

	Per et al. 2013 [23]	Tibussek et al. 2013 [24]	Değerliyurt et al. 2014 [25]	Bursztyn et al. 2014 [26]	Sheldon et al. 2016 [27]	Matthews et al. 2017 [28]	Mahajnah et al. 2020 [29]
1. Were the two groups similar and recruited from the same population?	Yes	Yes	Yes	Yes	Yes	Yes	Yes
2. Were the exposures measured similarly to assign people to both exposed and unexposed groups?	Yes	Yes	Yes	Yes	Yes	Yes	Yes
3. Was the exposure measured in a standard, reliable and valid way?	Yes	Yes	Yes	Yes	Yes	Yes	Yes
4. Were confounding factors identified?	Yes	Yes	Yes	Yes	Yes	Yes	Yes
5. Were strategies to deal with confounding factors stated?	Unclear	Yes	Unclear	Unclear	Yes	Unclear	Yes
6. Were the groups/participants free of the outcome at the start of the study (or at the moment of exposure)?	No	No	No	No	No	No	No
7. Were the outcomes measured in a reliable and valid way?	Yes	Yes	Yes	Yes	Yes	Yes	Yes
8. Was the follow-up time reported sufficient to be long enough for outcomes to occur?	Yes	Yes	Yes	N/A	N/A	Yes	N/A
9. Was follow-up complete, and if not, were the reasons for loss to follow-up described and explored?	Yes	Yes	Yes	Yes	Yes	Yes	Yes
10. Were strategies to address incomplete follow-up utilized?	N/A	N/A	N/A	N/A	N/A	N/A	N/A
11. Was appropriate statistical analysis used?	Unclear	Unclear	Yes	Yes	Yes	Yes	Unclear
12. Quality evaluation	Include	Include	Include	Include	Include	Include	Include

Actual Results

**Figure 3:**
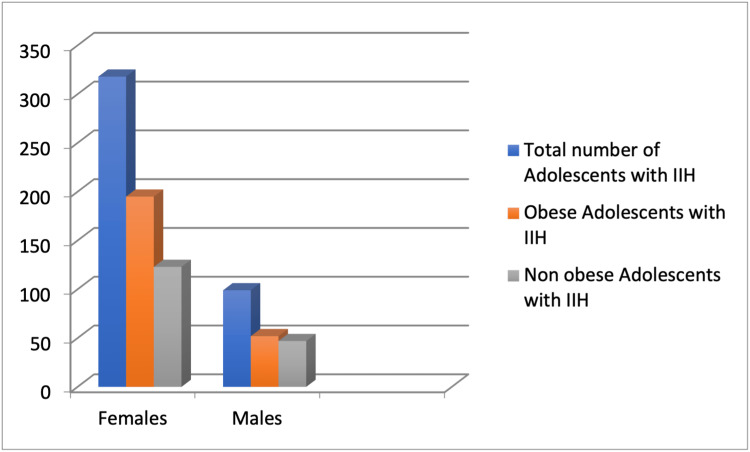
Data showing the distribution of obese female/male adolescents with IIH as compared to non-obese female/male adolescents with IIH IIH: idiopathic intracranial hypertension. Figure created by Sana Zafar.

Our systematic review gathered information from the 10 studies extracted after the quality check of the available limited data showing the association between obesity and IIH in adolescents. While all the studies showed the association mentioned above, the number of patients, criteria, and statistics for the studies varied. Across the selected studies, 417 adolescents were analyzed for this study topic, out of which 318 were females (76.3%) and 99 were males (23.7%), showing a greater propensity of the female population to have IIH. One hundred ninety-five of 318 females were obese (61.3%), and 52 of 99 males were obese (52.5%), making it more likely for obese female patients to have IIH than male adolescents, as shown in Figure 3.

Table 6 summarizes the results of these selected studies.

**Table 6:** The association of obesity and idiopathic intracranial hypertension in adolescents IIH: idiopathic intracranial hypertension, PTCS: pseudotumor cerebri syndrome. Ybarra et al., 2020 [20]; Brara et al., 2012 [21]; Stiebel-Kalish et al., 2014 [22]; Per et al., 2013 [23]; Tibussek et al., 2013 [24]; Değerliyurt et al., 2014 [25], Bursztyn et al., 2014 [26]; Sheldon et al., 2016 [27]; Matthews et al., 2017 [28];  and Mahajnah et al., 2020 [29].

Author and Year of Publication	Purpose of Study	Number of Patients/Study	Type of Study	Conclusion
Ybarra et al. (2020) [20]	To describe a case of an obese male with IIH treated with bariatric surgery	1	Case report	Bariatric surgery may be a valid treatment for morbidly obese refractory cases.
Brara et al. (2012) [21]	To estimate the magnitude of association between overweight, moderate, and extreme childhood obesity and IIH	66	Cross-sectional study	Childhood obesity is strongly associated with IIH, with extreme childhood obesity likely to lead to increased morbidity from IIH.
Stiebel-Kalish et al. (2014) [22]	To examine the hypothesis that being overweight or obese in adolescents increases the risk of IIH	29	Case-control study	A fivefold increase in the risk of IIH was noted in obese children compared to healthy controls.
Per et al. (2013) [23]	To estimate the etiological and clinical features of PTCS in children and adolescents	30	Retrospective study	PTCS is an avoidable cause of visual loss in children and adolescents; thus, early detection and management are important.
Tibussek et al. (2013) [24]	It aims to raise awareness of PTCS in pediatrics and contribute to a better understanding of age-related characteristics	29	Prospective study	Pediatric and adolescent PTCS is as frequent as in the general population.
Değerliyurt et al. (2014) [25]	To evaluate the clinical picture and etiological factors in adolescents	16	Retrospective study	PTCS is seen in prepubertal as well as after puberty, with increased incidence in obese adolescents.
Bursztyn et al. (2014) [26]	To understand if, like obesity, the incidence of IIH in children is rising? And it is related to that increase	9	Retrospective study	The result was a decreased incidence of IIH related to obesity which can be attributable to early diagnosis and intervention.
Sheldon et al. (2016) [27]	To study characteristics of diagnosis of IIH in adolescents	45	Retrospective study	Adolescents with IIH with increasing age are more likely to be obese.
Matthews et al. (2017) [28]	To investigate the epidemiology, clinical profile, and risk factors of PTCS in adolescents	152	Prospective study	Obesity is associated with IIH, and weight loss is central to the prevention of IIH.
Mahajnah et al. (2020) [29]	To study risk factors and clinical presentation of IIH	22	Retrospective survey	Risk factors in adolescents include obesity and female preponderance as in adults.

Discussion

Pseudotumor cerebri syndrome (PTCS), conveniently named idiopathic intracranial hypertension, has been revealed in children and adolescents since its publication in 1937 by Dandy [30]. Primary PTCS occurs in approximately 0.9 of 100,000 individuals in the general population, while its rate of incidence in young obese females has been revealed as 19.3 in 100,000 [31].

Diagnostic Criteria for IIH

The evolving definition of IIH, possibly due to advances in imaging technology, has been increasingly noticed in the last decade. According to the literature, this diagnosis can be established when the following criteria are met: (1) signs and symptoms pertinent to increased intracranial pressure (ICP) or papilledema; (2) increased ICP during lumbar puncture in lateral decubitus position; (3) normal CSF composition; (4) no evidence of enlarged ventricles or structural space-occupying lesions on imaging; and (5) no other source of increased pressure recognized such as medication use or venous sinus abnormality [1,8,10,32].

The words "benign intracranial hypertension" and "idiopathic intracranial hypertension" have been used interchangeably for a long time, but the word IIH cannot cater to all cases with PTCS. Based on the underlying cause, PTCS can be labeled as primary (no identifiable causative factor), secondary (identifiable cause), and atypical [33]. Some literature has used the words IIH and primary PTCS to depict the very same clinical syndrome. A set of "modified Dandy's criteria" was formulated after Dandy (in 1937) by Smith (in 1985) [34] and by Wall et al. (in 1991) [35], but the diagnostic criterion remains ambiguous [36,37]. Additional modifications to modified Dandy's criteria have been suggested owing to advances in neurologic and radiological investigations by Friedman and Jacobson [38] and for children by Rangwala and Liu [39] (Table 7). Ophthalmological examinations play an essential role in diagnosing IIH and neuroradiological investigations. Table 7 summarizes the diagnostic criteria.

**Table 7:** Diagnostic criteria for pediatric PTCS Adapted from Friedman and Jacobson, 2004 [38]. PTCS: pseudotumor cerebri syndrome, MRI: magnetic resonance imaging, CT: computed tomography, H_2_O: water, CSF: cerebrospinal fluid, WBC: white blood cells, MR venography: magnetic resonance venography, mm^3^: cubic millimeter, mg/dl: milligrams per deciliter. Table created by Sana Zafar.

Modified Dandy Criteria for Diagnosis of PTCS	Diagnostic Criteria Adapted From Rangwala for Pediatric PTCS
Signs and symptoms of increased intracranial pressure.	Prepubertal
No localizing findings on neurological examination.	Symptoms or signs of generalized intracranial hypertension or papilledema. Normal mental status
Normal MRI/CT brain scans with no central venous sinus thrombosis evidence.	Documented elevated intracranial pressure. Neonates: >76 mmH_2_O. Age less than eight with papilledema: >180 mmH_2_O
Increased intracranial pressure over 250 mmH2O and normal cerebrospinal fluid composition.	Eight years old or above, or less than eight years old without papilledema: >250 mmH_2_O
No other identified cause of intracranial hypertension	Normal CSF composition except in neonates who may have up to 32 WBC/mm^3^ and protein as high as 150 mg/dl
	No evidence of mass, structural, or vascular lesion or hydrocephalus on MRI, with and without contrast and MR venography. Narrowing of the transverse sinuses is allowed
	Cranial nerve palsies are allowed if they are of no other identifiable etiology and improve with a reduction in cerebrospinal fluid pressure or the resolution of other signs and symptoms of intracranial hypertension
	No other identified cause of intracranial hypertension

Clinical Features of IIH

The spectrum of clinical presentation of IIH or primary PTCS at the time of diagnosis is vast, which ranged from asymptomatic papilledema to substantial presentations including abducens nerve palsy, tinnitus, diplopia, or even stiff neck. Commonly reported symptoms include but are not limited to headache, nausea, photophobia, transient visual obscurations, anorexia, retro-orbital pain, lightheadedness, myalgia, head tilt, and eye movement abnormalities. The most frequently reported symptom in adolescents is a headache. Reports of chronic daily headaches or headaches worse in the morning that improves during the day were mainly noticed. After headache, diplopia or double vision was the second most frequent clinical feature reported. Vomiting and dizziness have also been observed to be associated with this condition.

The ophthalmological examination helps assess symptoms such as papilledema, visual field defects, reduced visual acuity, stereo and color vision disturbance, and eye movement disorders. Papilledema is generally considered a significant neurologic sign of PTCS, but the incidence of PTCS without papilledema has also been observed in some cases. In contrast to cases without papilledema, asymptomatic patients of PTCS have also been diagnosed by identifying papilledema in the routine ophthalmic examination. Periodic assessment of visual field defects has been helpful in the early detection of optic nerve damage. Permanent optic atrophy is a serious complication of untreated severe IIH cases. Neurologic studies have proven to be the essence in diagnosing cranial nerve paresis associated with IIH/PTCS. Facial nerve palsy is one of the unusual presentations reported with PTCS [39]. Lumbar puncture and cerebrospinal fluid analysis are also essential tools in diagnosing IIH.

Overweight/Obesity As a Risk Factor

In recent years, obesity has been a significant threat in childhood and adolescence. Overweight/obese adolescents have been shown to strongly associate with PTCS/IIH [22,36,40]. Overweight and obesity contribute to raised intracranial pressure by different mechanisms; reduced intracranial venous drainage might result from increased intrathoracic and intra-abdominal pressures, which may lead to decreased CSF absorption rate, eventually causing increased intracranial pressure [41]. It has also been observed that among obese patients for the reasons mentioned above, venous dysfunction is more common. Increased prevalence of obstructive sleep apnea (OSA) and elevated serum and CSF leptin levels among overweight patients might be another causative mechanism [42].

Furthermore, a strong association in the development of IIH has been observed with obesity. Children who tend to be overweight in childhood have been shown to have moderate/severe obesity, which leads to the development of a more severe nature of IIH with grave complications. In general, obese children tend to have accelerated growth before puberty and may have lesser growth during puberty. There is a possibility that the factors such as growth hormones that affect linear growth status in overweight/obese adolescents may influence the development of increased intracranial pressure. Excess gonadal hormone secretion during puberty might play a role in the pathogenesis of PTCS [28]. Adolescent patients with marked obesity have been associated with having a fivefold increased risk of developing a worse clinical picture of PTCS [24].

Treatment and Complication/Prognosis

Treatment for PTCS aims to decrease intracranial pressure, which can be achieved with medical or surgical therapy. The goal of treatment is to relieve symptoms by controlling CSF pressure to avert vision loss. In the medical treatment of PTCS, the drug of choice is the carbonic anhydrase inhibitor, acetazolamide, which acts by decreasing CSF production. After acetazolamide, topiramate, an antiepileptic drug, works by inhibiting carbonic anhydrase enzyme and reduces CSF production. Topiramate has additional benefits of analgesia and weight loss [25]. Other medical treatment options include furosemide and prednisone [22,39,43]. Steroids like prednisone reduce intracranial pressure efficiently and are highly recommended for parenteral use or short-term oral treatment only in severe headaches, severe papilledema, and very high intracranial pressure. However, they are generally not recommended for chronic treatments due to their side effect profile of weight gain and increased intracranial pressure in the rebound period.

Surgical treatment options are advised for refractory cases, including repeated lumbar punctures and ventriculoperitoneal shunting to drain excess CSF and normalize CSF pressure. Optic nerve sheath fenestration has also been implicated in refractory cases of papilledema to prevent permanent optic nerve damage. Weight loss strategies have also been seen to improve cases of PTCS. Calorie deficit diets and medical weight loss strategies are recommended. Some cases may require bariatric surgery to help accelerate weight loss and prevent short-term and long-term consequences of obesity-associated PTCS [21,44,45].

Complications of prolonged increased intracranial pressure include optic atrophy which causes permanent visual field constriction and vision loss [7,24]. Recurrence of IIH is another feared complication of this spectrum of clinical diseases in obese adolescents. According to our review, the long-term prognosis of this condition depends on the obesity status of adolescents along with the response to other treatment modalities. Resolution of cases has been reported in the literature with early diagnosis, prompt treatment, and compliance to treatment [21,23,25,26].

Limitations

This systematic review, which consists of 10 published papers, has limitations like the count of study participants were all different, such as Matthews et al. [28], with a sample count of 152 participants, compared to Bursztyn et al. [26], with only nine participants. Furthermore, as per our eligibility criteria, which riveted on papers published in English or literature which had English translation published within the last 10 years only, focusing on adolescent age group participants, it may have resulted in the exclusion of several papers owing to not being able to qualify our inclusion/exclusion criteria.

## Conclusions

We aimed to explore a significant association between idiopathic intracranial hypertension and adolescent obesity. According to the reviewed literature, IIH/PTCS is a clinical entity that affects adolescents, especially childbearing age, female adolescents. Obesity has been seen to play a massive role in developing IIH/PTCS. Early diagnosis with periodic neuro-ophthalmologic examinations of IIH in adolescents is essential to prevent permanent visual impairment. The physicians should be familiar with the varied presentations of PTCS along the lifespan and keep a high index of suspicion to prevent missed diagnosis. They should also advise families of weight control strategies to help prevent the dire outcomes of this disease.
